# Characterization and comparison of temperature-sensitive *Schizosaccharomyces pombe* mutants of the septation initiation network scaffolds, Cdc11 and Sid4

**DOI:** 10.17912/micropub.biology.001503

**Published:** 2025-02-14

**Authors:** Lesley A. Turner, Anna Bowman Fletcher, Alaina H. Willet, Kathleen L. Gould

**Affiliations:** 1 Department of Cell and Developmental Biology, Vanderbilt University School of Medicine, Nashville, TN, US

## Abstract

The *Schizosaccharomyces pombe* septation initiation network (SIN) is required for cytokinesis and septation. The SIN includes a protein kinase cascade that is assembled at spindle pole bodies (SPBs) in a cell cycle specific manner on a scaffold consisting of Cdc11 , related to human centriolin, and the a-helical protein Sid4 . Here, we characterized temperature-sensitive *cdc11* and *sid4* mutants isolated in the 1990s. We determined the mutations within each allele, examined their phenotypes, and analyzed their growth compared with previously characterized mutant alleles. The new mutants described here expand the toolkit for studying how the SIN assembles at SPBs.

**Figure 1. Characterization of sid4 and cdc11 mutant alleles.**
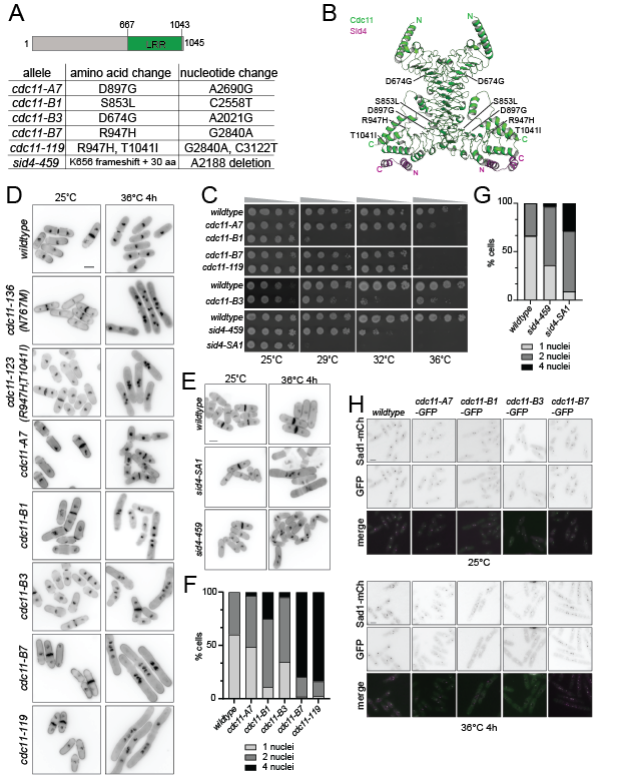
(A) Schematic of Cdc11 , drawn to scale, with the predicted leucine-rich repeat (LRR) domain indicated in green. The *cdc11* and *sid4* mutations encoded by each allele are listed. (B) AlphaFold3 predicted structure and interaction between two copies of Cdc11 (residues 571-1045) in green and Sid4 (residues 41-72) in magenta. The residues mutated in the *cdc11* alleles are labelled. (C) The indicated strains were grown in liquid YE at 25°C until they reached mid-log phase and then adjusted to the same cell concentration measured by optical density (Moreno et al., 1991). Next, 10-fold serial dilutions were prepared and 2.5 µL of each was spotted on YE agar plates and incubated at the indicated temperatures for 2-5 days prior to imaging. (D-E) The indicated strains were grown at 25˚C and shifted to 36˚C for 4 hours. Samples were collected at both temperatures and cells were fixed in 70% ethanol and stained with DAPI and methyl blue before imaging. Scale bar, 5 µm. (F-G) Quantification of the number of nuclei per cell of the indicated strains from D and E. Scale bar, 5 μm. (H) Representative live-cell images of the indicated strains grown at 25˚C (top panels) and then shifted to 36˚C for 4 hours (bottom panels). Scale bar, 5 μm.

## Description

*Schizosaccharomyces pombe* spindle pole bodies (SPBs) serve as cellular signaling platforms (Cavanaugh and Jaspersen, 2017) . One signaling pathway that is assembled at SPBs is the septation initiation network (SIN) (reviewed in (Johnson et al., 2012; Simanis, 2015; Xiao and Dong, 2021) . The SIN is a GTPase-regulated protein kinase cascade required in *S. pombe* for the formation, maintenance, and constriction of the actomyosin-based cytokinetic ring; the SIN also guides the recruitment and function of cell wall enzymes necessary for septation (Cheffings et al., 2016; Glotzer, 2017; Mangione and Gould, 2019) . Thus, in SIN mutants, cell division fails.

SIN signaling proteins are recruited by the centriolin-like scaffold protein, Cdc11 , which is stably tethered to the SPB by the ɑ-helical protein, Sid4 (Chang and Gould, 2000; Krapp et al., 2004; Krapp et al., 2001; Morrell et al., 2004; Tomlin et al., 2002). Sid4 , in turn, is anchored to the core *S. pombe* SPB protein Ppc89 (Hanna et al., 2024) .

In a genetic screen for cytokinesis factors, several mutants mapping to *cdc11* and a second mutant of *sid4* were isolated but not previously characterized (Balasubramanian et al., 1998; Nurse et al., 1976) . We first determined the mutations within these five alleles and also in *cdc11-119* that was not previously reported in PomBase (Rutherford et al., 2024) . For this, the *cdc11* or *sid4* open reading frame (ORF) was amplified from each strain and sequenced. The *cdc11-119* allele contained the same mutations as previously described for *cdc11-123* (R947H and T1041I) (Rutherford et al., 2024) ( Figure 1A ). *cdc11-B7* had one of these mutations (R947H)( Figure 1A ). All mutations identified in the various *cdc11* alleles, including the previously sequenced *cdc11-136* allele fall within the leucine-rich repeat (LRR) domain of the protein (Rutherford et al., 2024) ( Figure 1A-C ). AlphaFold3 (Abramson et al., 2024) predicted that the Cdc11 LRRs form a parallel dimer so that both sets of N- and C-termini are positioned at opposite ends of the predicted structure ( Figure 1B ). Further, when modelled with two N-termini of Sid4 , AlphaFold3 predicted a direct interaction, as expected from previous structure-function analysis (Krapp et al., 2004; Tomlin et al., 2002) . The predicted binding interface consists of Sid4 residues 42-71 which form a pair of ɑ-helices that each dock onto the very C-terminus of a Cdc11 LRR molecule ( Figure 1B ). The residues that are mutated in the various *cdc11* alleles map near the Sid4- Cdc11 binding interface or the Cdc11-Cdc11 dimer interface, suggesting that these interactions could be disrupted in the mutants ( Figure 1B ). Unexpectedly, no mutations were identified in the unstructured N-terminus of Cdc11, the region of the protein required to recruit several SIN components to the SPB (Krapp et al., 2004) . The mutation in *sid4-459* resulted in a frameshift mutation that causes a loss of the last four amino acids of Sid4 and the addition of 30 nonsense amino acids to the protein. The C-terminus of Sid4 docks to the SPB scaffold, Ppc89 (Hanna et al., 2024,) and therefore Sid4-459 likely loses its Ppc89 connection at restrictive temperature as does the mutation in Sid4-SA1 (L629P) (Chang and Gould, 2000) .

To characterize the new mutants, we first compared their growth to wildtype cells and the previously sequenced and characterized alleles ( *cdc11-136* and *cdc11-123; sid4-SA1* ). While all strains grew at 25°C, the temperature sensitive alleles grew poorly or not at all at 36°C ( Figure 1C ). To visualize the cell phenotypes, we stained the nuclei and septa after the cells were grown at 25°C and then shifted or not to 36˚C for 4 hours. At 25°C, all strains resembled wildtype ( Figure 1D-E ). Upon shift to the restrictive temperature, most *cdc11-136* , *cdc11-123* , *cdc11-B7* and *cdc11-119* cells became multinucleated and did not form a septum ( Figure 1D and F). *cdc11-B1* and *cdc11-B3* cells also became binucleated or multinucleated, but some appeared to attempt septation and failed ( Figure 1D and F). Lastly, *cdc11-A7* and *sid4-459* cells primarily formed a “boomerang” of paired mononucleate cells that frequently lysed ( Figure 1D-F ). These latter phenotypes are indicative of less penetrant SIN mutants.

Lastly, we examined the localization of the Cdc11 mutant proteins by tagging each with GFP. It was previously reported that while Cdc11-123-GFP is lost from the SPB at restrictive temperature, Cdc11-136-GFP remains there indicating that 1) it retains its ability to bind Sid4 , and 2) it loses its ability to recruit a downstream SIN component (Krapp et al., 2001) . Cdc11-A7, like Cdc11-136, was retained at the SPB at 36˚C while Cdc11-B1 SPB localization was lost and Cdc11-B3 was reduced ( Figure 1H ). Interestingly, we found that Cdc11-B7 remained at the SPB at 36˚C. This mutation shares the R947H mutation with Cdc11-123 that is lost from the SPB, indicating that T1041 is involved in tethering Cdc11 to Sid4 and that R947 must be involved in a distinct interaction ( Figure 1H ). Taken together, the characterization of these *cdc11* mutants has informed the separable functions of the Cdc11 LRR and it will be interesting in future studies to examine what interaction(s) Cdc11 residues 897 and 947 are involved in.

## Methods

Yeast strains and methods

*S. pombe* strains were grown in yeast extract (YE) and standard *S. pombe* mating, sporulation, and tetrad dissection techniques were used to construct new strains (Moreno et al., 1991) . All spot assays were performed twice with reproducible results.

Tagged strains were generated by adding sequences encoding green fluorescent protein (GFP) and resistance cassette *kanMX6* or mCherry and resistance cassette *natMX6* at the 3′ end of the endogenous open reading frame using pFA6 cassettes as previously described (Bahler et al., 1998; Wach et al., 1994) . G418 (geneticin, 100 µg/mL, Thermo Fisher Scientific; cat# 11811031) and nourseothiricin (clonNAT, 100 µg/mL, GoldBio; cat# N-500-100) were used for selection of *kanMX6* or *natMX6* cells, respectively. Successful tagging of the strains was verified by whole cell PCR (forward oligos: a 20 bp sequence located ~200 bp upstream of the stop codon of the corresponding ORF; reverse oligos: CGCTTATTTAGAAGTGGCGCG, which is a common sequence in the *adh1* terminator present in the pFA6 cassettes, TCATCCATGCCATGTGTAATCC, for GFP, and GTACAGTCTGTCCATGCCGC for mCherry).

Molecular biology methods

The *cdc11* open reading frame from *cdc11-B3, cdc11- B7,* and *cdc11-119* cells was amplified using an oligonucleotide 74 bp upstream of the start site (GATTGAGTCCCAGTACCACG) and a second oligonucleotide 45 bp downstream of the stop codon (CAACAGCGAAACAATCTTGCT) (Integrated DNA technologies). The *cdc11* open reading frame was amplified from *cdc11-A7* and *cdc11-B1* cells using overlapping oligonucleotides. Specifically, an oligonucleotide 351 bp upstream of the start site (GTGAATCTCTCATGCACAAG), an oligonucleotide within the ORF (CTAGCATCTTCGTCGGTTTCA), as well as another oligonucleotide within the ORF at 1500 bp (CCTCATTCCTTTCCTTTGCGT) and an oligonucleotide 695 bp downstream of the stop codon (TCGTTCTCTGTCTTCCTATG) were used (Integrated DNA technologies). The *sid4* open reading frame was amplified using an oligonucleotide 64 bp upstream of the start site (CGAGCATGTGACTTACACTC) and a second oligonucleotide 94 bp downstream of the stop codon (ACGCCTCTTTCATTCAGTCAG) (Integrated DNA technologies). The PCR products were each sequenced using Oxford Nanopore Technology with custom analysis and annotation (Plasmidsaurus).

Microscopy and image analysis

Strains for fixed-cell and live-cell imaging experiments were grown at 25°C in YE and then shifted to 36°C for 4 hours. Cells were fixed with 70% ethanol for DAPI and methyl blue (MB) staining as described previously (Roberts-Galbraith et al., 2009). Images were acquired using a Zeiss Axio Observer inverted epifluorescence microscope with Zeiss 63× oil objective (1.46 NA) and captured using Zeiss ZEN 3.0 (Blue edition) software. For fixed-cells, a singular medial Z slice was obtained. For live-cells, images were acquired with a z-stack step size of 0.50 µm and a total of 10 z-slices. All images were further processed using ImageJ (Schindelin et al., 2012) . Live-cell representative images were deconvolved and projected with average intensity. Images used for all imaging experiments were repeated twice.

AlphaFold3 structural prediction

Protein structure predictions were generated with the AlphaFold3 server (Abramson et al., 2024) and visualized using the PyMOL molecular graphics system (version 3.0, Schrodinger, LLC).

## Reagents

The strains used in this study and their genotypes are listed below.

**Table:** 

**Strain**	**Genotype**	**Source**	
KGY101	* cdc11-136 h ^-^*	*Nurse et al., 1976*	
KGY69-2	* cdc11-A7 ura4-D18 h ^+^*	This study	
KGY103	* cdc11-123 h ^-^*	*Nurse et al., 1976*	
KGY107	* cdc11-119 h ^-^*	*Nurse et al., 1976*	
KGY113-2	* cdc11-B1 ura4-D18 h ^-^*	This study	
KGY246	* ade6-M210 ura4-D18 leu1-32 h ^-^*	This study	
KGY281-2	* cdc11-B7 ura4-D18 h ^-^*	This study	
KGY639-2	* cdc11-GFP:KanMX6 sad1-mCherry:NatMX6 ade6-M210 leu1-32 ura4-D18 h ^-^*	This study	
KGY718-2	* cdc11-B3-GFP:KanMX6 sad1-mCherry:NatMX6 ura4-D18 h ^+^*	This study	
KGY841-2	* cdc11-A7-GFP:KanMX6 sad1-mCherry:NatMX6 ura4-D18 h ^-^*	This study	
KGY825-2	* cdc11-B1-GFP:KanMX6 sad1-mCherry:NatMX6 ura4-D18 h ^+^*	This study	
KGY1033	* cdc11-A7 ura1 leu1-32 mam2 ::LEU2 ade6-M216 h ^90^*	*Balasubramanian et al., 1998*	
KGY1034	* cdc11-B1 ura1 leu1-32 mam2 ::LEU2 ade6-M216 h ^90^*	*Balasubramanian et al., 1998*	
KGY1036	* cdc11-B3 ura1 leu1-32 mam2 ::LEU2 ade6-M216 h ^90^*	*Balasubramanian et al., 1998*	
KGY1039	* cdc11-B7 ura1 leu1-32 mam2 ::LEU2 ade6-M216 h ^90^*	*Balasubramanian et al., 1998*	
KGY1234	*sid4-SA1 ura4-D18 leu1-32 ade6-M210 h-*	Lab stock	
KGY1742-2	*cdc11-B7-GFP:KanMX6 sad1-mCherry:NatMX6 ura4-D18 h-*	This study	
KGY2746	*sid4-459 leu1-32 ura4-D18 his3-D1 h-*	*Balasubramanian et al., 1998*	
KGY10010-2	*cdc11-B3 ura4-D18 h+*	This study	
